# Redescriptions of Two Tintinnid Ciliates (Ciliophora: Tintinnida) from Freshwater Lake in China Based on Living Morphology and Ciliary Pattern, with a Comparison to Related Species

**DOI:** 10.3390/ani16081197

**Published:** 2026-04-14

**Authors:** Han Fang, Yuxuan Yin, Yajuan Li, Yang Bai

**Affiliations:** 1 Inner Mongolia Key Laboratory of River and Lake Ecology, School of Ecology and Environment, Inner Mongolia University, Hohhot 010021, China; fh20020909@163.com (H.F.); zjk16719987@163.com (Y.Y.); 2 Collaborative Innovation Center for Grassland Ecological Security (Jointly Supported by the Ministry of Education of China and Inner Mongolia Autonomous Region), Hohhot 010021, China; 3 College of Animal Science & China-Mongolia Biomacromolecule Application “Belt and Road” Joint Laboratory, College of Animal Science, Inner Mongolia Agricultural University, Hohhot 010018, China

**Keywords:** ciliary pattern, freshwater tintinnid, living observation, lorica, *Tintinnopsis*

## Abstract

As a dominant group of microzooplankton, the tintinnid ciliates (Ciliophora: Tintinnida) play a crucial role in aquatic ecosystems by linking the microbial food loop with the classical planktonic food web. However, taxonomic understanding has been greatly hindered by lorica polymorphism, combined with limited data on living cells and infraciliature, which in turn constrains progress in related fields such as ecology and physiology. In the present study, two freshwater tintinnids, namely *Tintinnopsis wangi* Nie, 1933, and *T. tubuformis* Chiang, 1956, collected from the Weishan Lake Basin, China, were redescribed based on living observation and protargol staining. Furthermore, the ciliary pattern of *T. wangi* is first reported, while that of another species corresponds well with its Shanghai population, indicating relatively high intraspecific stability of ciliary patterns in Tintinnida. In addition, based on the comparison of morphological characteristics, *T. anhuiensis* Nie, 1933, and *T. sinensis* Chiang, 1956, are considered synonyms of *T. wangi*. The characteristics of all species were compared with their original descriptions and further studies.

## Simple Summary

Microscopic aquatic organisms are vital components of freshwater ecosystems, yet accurately identifying specific groups, such as tintinnids, remains challenging. Historically, identification relied solely on their diverse lorica, which can change shape and lead to misclassification. This study conducted a detailed morphological study on two species of freshwater tintinnids collected from a lake in China. The results revealed the previously unknown ciliary pattern of one species and confirmed the structural stability of the other. Furthermore, this study found that among the currently known freshwater ciliates with a ciliary pattern, species possessing a posterior kinety are all composed of monokinetids, which clearly differs from marine ciliates. Accurately documenting information about these microscopic organisms is crucial for monitoring water environment changes, as they are highly sensitive to environmental variations. This enhanced understanding provides a more reliable foundation for monitoring water quality, evaluating aquatic health, and supporting biodiversity conservation efforts in freshwater environments.

## 1. Introduction

As a key numerical component of microzooplankton, planktonic ciliates are recognized as pivotal contributors to the microbial food loops for aquatic ecosystems such as oceans, lakes, and rivers, forming an essential trophic link between primary producers and higher-level consumers [1,2,3,4,5]. Among these, tintinnid ciliates are characterized by their highly diverse loricae and have attracted considerable attention due to several factors: (1) their community structure and geographical distribution exhibit distinct patterns, providing important cases for biogeographical studies [6,7,8]; (2) they are sensitive to environmental changes and can serve as bioindicators for water quality assessment and tracing hydrological circulation [9,10]; (3) within food webs, they provide a food source for fish larvae and other small planktonic metazoans [11,12]; and (4) their loricae can be preserved as fossil records, offering valuable materials for evolutionary biology research [13,14].

To date, approximately 1200 nominal tintinnid species have been described, with the majority of species identifications (over 90%) relying solely on morphological characteristics of their loricae [15,16,17,18]. However, the laboratory culture of a limited number of species have demonstrated that their lorica morphology had obvious polymorphism within the same species under both natural and cultured conditions [19], highlighting the limitations of using lorica features alone for accurate taxonomic classification [20]. In response, Santoferrara et al. [21] proposed updated identification protocols for loricate protists, emphasizing the necessity of integrating lorica morphology and cytological traits to advance research on tintinnids. Encouragingly, efforts to combine these approaches have increased in recent years [4,22,23,24,25,26,27]. Despite this progress, comprehensive data on ciliary structures remain available for only about 5% of described tintinnid morphospecies [20]. Subsequently, relevant studies have focused primarily on marine habitat, and merely eight freshwater species have their ciliary patterns documented [28,29,30,31], which underscore significant gaps, particularly within freshwater groups, in the taxonomic characterization of tintinnids.

The genus *Tintinnopsis* was originally erected by Stein [32], with *Tintinnopsis beroidea* as its type species. After more than a century of research, over 140 species have been described to date, making it the most species-rich genus within the order Tintinnida [16,17,18]. Due to insufficient taxonomic studies, their systematics developed slowly.

In the present study, two freshwater tintinnid ciliates, namely *Tintinnopsis wangi* Nie, 1933, and *T. tubuformis* Chiang, 1956, collected from a fishpond of Weishan Lake, China, were morphologically examined based on live and protargol-stained materials. This study aims to expand the database and understanding about the diversity of this group of eukaryotic microorganisms.

## 2. Material and Methods

**Figure 1:**
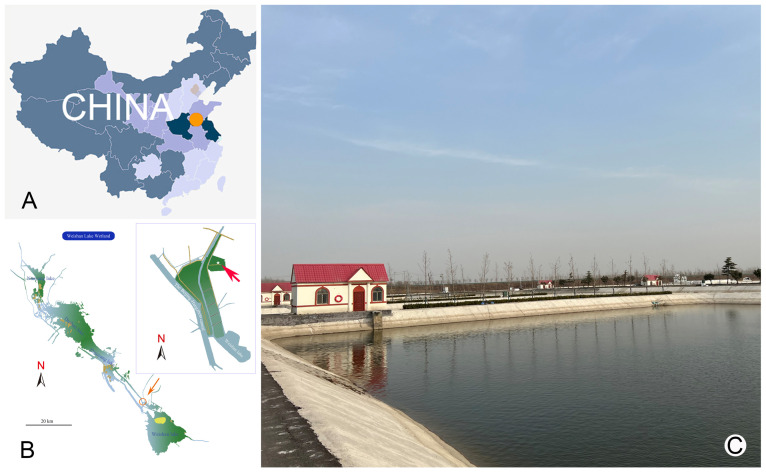
(**A**) Map and photographs of the localities. (**A**,**B**) Map of China and Weishan Lake, where the orange cycle and red arrow indicate the sampling site. (**C**) Photograph of the sampling site.

All samples were collected on 20 February 2023 from the surface water (0–2 m depth) of a fishpond in the Weishan Lake Wetland, China (34°46′14″ N, 117°12′56″ E), using a plankton net with a mesh size of 25 μm (Figure 1A–C). The water samples were deposited in Petri dishes (200 mm in diameter, approximately 5 mm deep) and maintained at room temperature (25 °C) prior to processing. The cells were isolated under a stereo microscope (Olympus SZX2-TR30, Tokyo, Japan) at 45× magnification using micropipettes. Observations of live cells were carried out employing bright-field and differential interference contrast microscopy (Olympus BX53, Tokyo, Japan) across a magnification range of 100–1000×. Lorica dimensions were measured from living cells at magnifications of 100–400×. Subsequent to fixation in Bouin’s solution, an eyebrow brush was used to dissociate the cell body from the lorica. With the powder manually synthesized as per Pan et al. [33], protargol staining following Wilbert’s [34] protocol enabled observation of the infraciliature and nuclear apparatus. All measurements and camera lucida drawings of protargol-impregnated specimens were performed under 1000× magnification. Taxonomic identification was conducted with reference to original descriptions and the established tintinnid literature, while the terminology and systematics adopted are those of Agatha and Riedel-Lorjé [35] and Santoferrara et al. [36], respectively.

## 3. Results

**Phylum Ciliophora Doflein, 1901**

**Class Oligotrichea Bütschli, 1887**

**Subclass Choreotrichia Small & Lynn, 1985**

**Order Tintinnida Kofoid & Campbell, 1929**

**Genus *Tintinnopsis* Stein, 1867**

**Figure 2:**
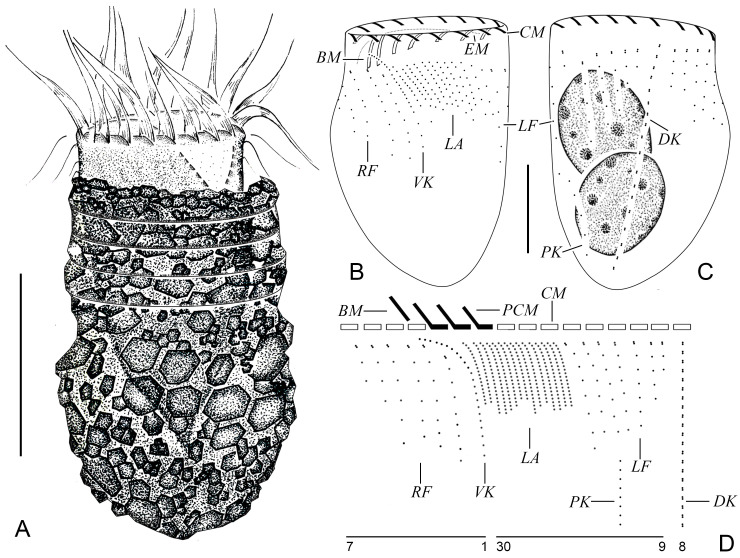
Specimens of *Tintinnopsis wangi* Nie, 1933, in vivo (**A**) and after protargol staining (**B**–**D**); (**A**) lateral view of a representative individual. (**B**,**C**) Ventral (**B**) and dorsal (**C**) views of the same specimen; (**D**) kinetal map of a morphostatic specimen. BM, buccal membranelles; CM, collar membranelles; DK, dorsal kineties; EM, endoral membrane; LA, lateral ciliary field; LF, left ciliary field; PCM, prolonged collar membranelles; PK, posterior kineties; RF, right ciliary field; VK, ventral kinety. Scale bars: 30 μm (**A**), 15 μm (**B**,**C**).

**Figure 3:**
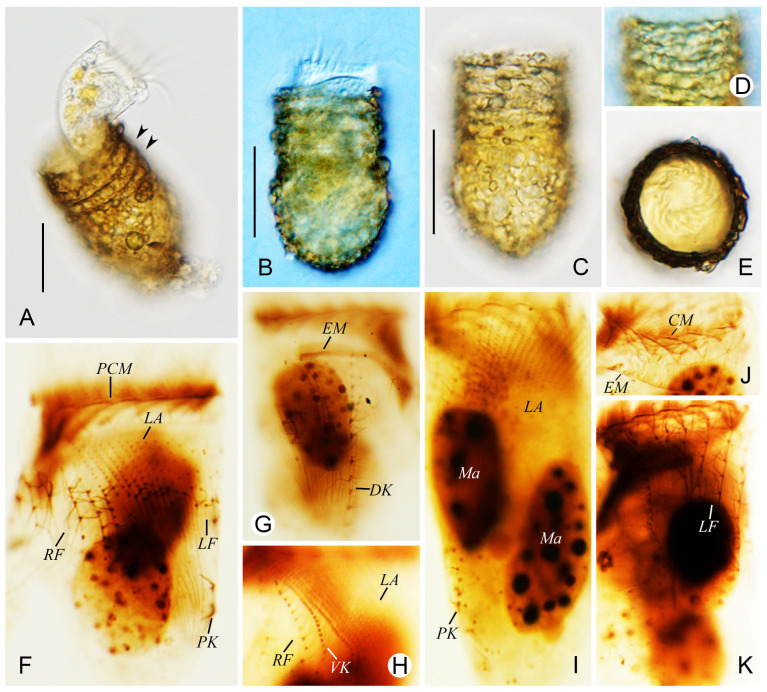
Photomicrographs of *Tintinnopsis wangi* Nie, 1933, in vivo (**A**–**E**) and after protargol staining (**F**–**K**). (**A**) Lateral view of a representative individual, where arrowheads indicate the annular striaes. (**B**) Cell body extends from lorica. (**C**) Lorica surface with agglutinated particles. (**D**) Annular striae. (**E**) Apical view, showing the collar membranelles. (**F**,**G**) Ventral (**F**) and dorsal (**G**) views of the same individual after protargol staining. (**H**) Ventral kinety, right and lateral ciliary field. (**I**) Lateral ciliary field and posterior kinety. (**J**) Collar membranelles and endoral membrane. (**K**) Left ciliary field. CM, collar membranelles; DK, dorsal kinety; EM, endoral membrane; LA, lateral ciliary field; LF, left ciliary field; Ma, macronucleus; PCM, prolonged collar membranelles; PK, posterior kinety; RF, right ciliary field; VK, ventral kineties. Scale bars: 30 μm (**A**–**C**).

***Tintinnopsis wangi* Nie, 1933** (Figure 2 and Figure 3; Table 1)

**Improved diagnosis about *Tintinnopsis wangi* Nie, 1933 (based on original description and this study).** Lorica is vase-shaped, comprising a cylindrical collar and an ovoid bowl, 42–71 μm long and 32–40 μm wide. Collar possesses 3–6 annular constrictions, with opening 27–34 μm in diameter. Two macronuclear nodules. The collar membranelles number on average 17, comprising three that extend into the buccal cavity, in addition to a single buccal membranelle. The ventral kinety comprises approximately 20 monokinetids, while the right, lateral, and left ciliary fields contain about nine, 15, and eight kineties, respectively. The dorsal kinety consists of about 19 dikinetids, whereas the posterior kinety is composed of around 6 monokinetids.

**Deposition of neotype material.** A protargol slide containing the neotype specimen was deposited in the Laboratory of Protozoology, Institute of Evolution and Marine Biodiversity, Ocean University of China, with the registration number BY202302200101.

**Habitat.** Water temperature 9 °C.

**Zoobank registration:** LSID: F4063D27-18FD-433D-9518-12CB8A160D32.

**Morphological description.**

The vase-shaped lorica measured 42–71 μm in length, comprising a cylindrical collar and an ovoid bowl, with an opening 27–34 μm in diameter (Figure 2A and Figure 3A–D). The collar, which accounted for 1/3–1/2 of the total length and displayed 3–6 annular constrictions, had a wall that was parallel to the central axis (Figure 2A and Figure 3A–E). The convex bowl measured 32–40 μm in width at its broadest (anterior) part and narrowed gradually posteriorly (Figure 2A and Figure 3A–D). Posterior end usually rounded to bluntly tapered (Figure 2A and Figure 3A–D). Densely packed mineral particles of various sizes were agglomerated on the lorica wall (Figure 2A and Figure 3A–D).

When fully extended, the living cell body measured approximately 40–60 × 20–35 μm and 47–72 × 27–37 μm after protargol staining (Figure 2A and Figure 3A). The posterior region of the cell tapered gradually into a peduncle approximately 10–20 μm in length, which was anchored to the base of the lorica. Two ovoid macronuclei, each about 15–19 μm long and 10–14 μm wide after protargol staining (Figure 2C and Figure 3I). Micronuclei, striae, tentaculoids, accessory combs, contractile vacuole, cytopyge, and capsules not observed. A colorless and transparent cytoplasm, with food vacuoles commonly containing microalgae or unidentified particles (Figure 2A and Figure 3A). While rotating forward around its central axis in water, the cell often had one-fifth to one-quarter of its body extended out of the lorica.

Somatic ciliary pattern consisted of ventral, dorsal, and posterior kineties and right, left, and lateral ciliary fields (Figure 2B–D and Figure 3F–K). The ventral kinety commenced above the second kinety of the right ciliary field, 1–2 μm posterior to the collar membranelles, curved around the right ciliary field from the left side and extended posteriorly parallel to the lateral ciliary field; 17–26 μm in length, composed of 16–24 densely arranged monokinetids (Figure 2B,D and Figure 3F,H). The kineties of the right ciliary field numbered 7 to 11, spaced 2–5 μm apart from each other.; most kineties originated about 5–10 μm posterior to the collar membranelles, while the first kinety began about 2 μm posterior to the others; each kinety comprised approximately 2–10 widely spaced monokinetids and a single anterior dikinetid (Figure 2B–D and Figure 3F,H). The left ciliary field comprised 7–9 kineties, with an interkinety distance of about 2–5 μm; originating approximately 5–10 μm posterior to the collar membranelles, each kinety composed of about 1–12 widely spaced monokinetids and a single anterior dikinetid (Figure 2B–D and Figure 3F,K). In protargol-stained specimens, each basal body in the ciliary fields possessed a cilium, with the anterior cilium of each dikinetid measuring about 10 μm in length, whereas all other cilia (from monokinetids and the posterior cilium of dikinetids) were approximately 5 μm long (Figure 3F,G,K). Lateral ciliary field composed of 12–17 closely arranged monokineties with similar length; cilia about 2–5 μm after protargol staining (Figure 2B,D and Figure 3F,H,I). The dorsal kinety, 37–52 μm in length and composed of 16–24 dikinetids, originated approximately 2 μm posterior to the collar membranelles and about 5 μm from the right and left ciliary fields, respectively; after protargol staining, only the posterior basal body of each dikinetid bore a cilium about 10 μm long (Figure 2C,D and Figure 3G). Posterior kinety 10–15 μm long, consisted of 5–7 sparsely arranged monokinetids with a long cilium on each monokinetid about 10 μm after protargol staining, commenced posterior to the second to 4th kineties of the left ciliary field (Figure 2C,D and Figure 3F,I).

Oral apparatus occupying anterior portion of cell (Figure 2A and Figure 3E). The adoral zone of membranelles formed a closed loop composed of 16–18 collar membranelles, three of which extended into the buccal cavity; the polykinetids measured approximately 15 μm in length (Figure 2B–D and Figure 3G). Cilia of membranelles about 15–25 μm long (Figure 2A and Figure 3B). A single buccal membranelle, approximately 10 μm long at its base, was located within the buccal cavity. The endoral membrane consisted of a single row of basal bodies, curving around the dorsal side of the buccal cavity and extending toward the right side (Figure 2B and Figure 3G,J).

**Neotypification**.

A neotype of *Tintinnopsis wangi* is designated for the following reasons: (1) no type material is known to have been preserved; (2) the original species description relies solely on lorica morphology; (3) the genus *Tintinnopsis* is currently recognized as paraphyletic; and (4) Nanjing, the type locality of the initial population, is situated near Weishan Lake, China [37], and therefore satisfies the requirement of Article 75.3.6 of the International Code of Zoological Nomenclature, which states that “the neotype came as nearly as practicable from the original type locality” [38]. Above a full description of the new type locality is presented, i.e., the sampling site from which the neotype population was obtained.

A protargol slide of the neotype specimen, along with a slide carrying voucher specimens, has been deposited in the collection of the Ocean University of China (see ‘Deposition of neotype material’ for details). This deposition satisfies the stipulations of Article 75.3.7 of the ICZN [38].

**Figure 4:**
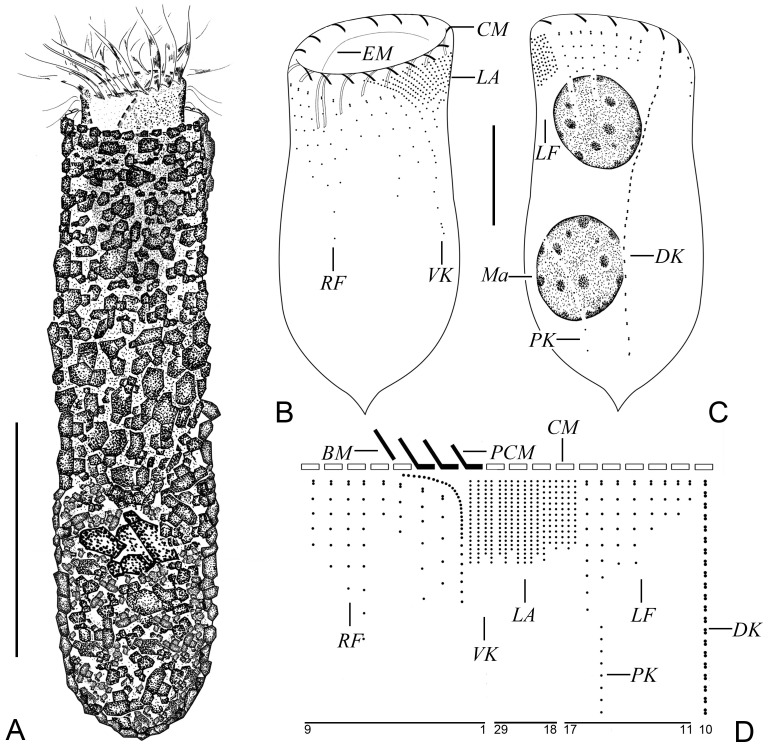
Specimen of *Tintinnopsis tubuformis* Chiang, 1956, in vivo (**A**) and after protargol staining (**B**–**D**); (**A**) lateral view of a representative individual. (**B**,**C**) Ventral (**B**) and dorsal (**C**) views of the same specimen; (**D**) kinetal map of a morphostatic specimen. BM, buccal membranelles; CM, collar membranelles; DK, dorsal kineties; EM, endoral membrane; LA, lateral ciliary field; LF, left ciliary field; PCM, prolonged collar membranelles; PK, posterior kineties; RF, right ciliary field; VK, ventral kinety; Ma, macronucleus. Scale bars: 35 μm (**A**), 20 μm (**B**,**C**).

**Figure 5:**
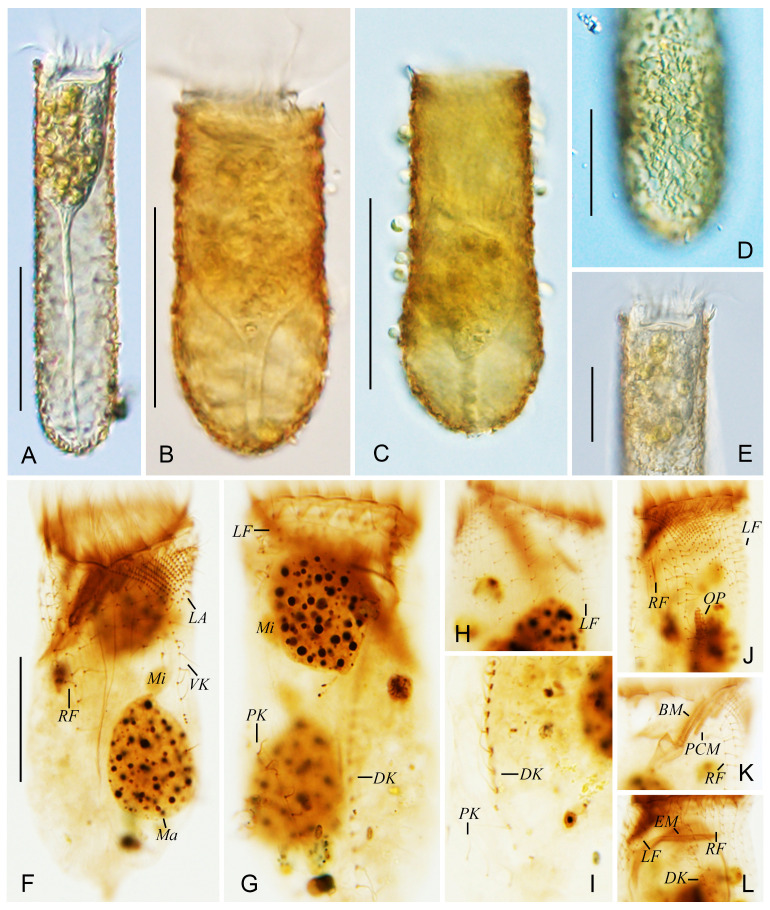
Photomicrographs of *Tintinnopsis tubuformis* Chiang, 1956, in vivo (**A**–**E**) and after protargol staining (**F**–**I**). (**A**) Lateral view of a representative individual. (**B**,**C**) Individuals with different lorica morphologies. (**D**) The lorica surface of posterior end. (**E**) Anterior portion of lorica. (**F**,**G**) Ventral (**F**) and dorsal (**G**) views of the same individual after protargol staining. (**H**) Left ciliary field. (**I**) Dorsal kinety and posterior kinety. (**J**) Ventral view of an early divider, showing oral primordium. (**K**) Buccal membranelle and prolonged collar membranelles. (**L**) Dorsal view of a middle divider, showing the fragmentations of dorsal kinety. BM, buccal membranelle; DK, dorsal kinety; EM, endoral membrane; LA, lateral ciliary field; LF, left ciliary field; Ma, macronucleus; Mi, micronucleus; OP, oral primordium; PCM, prolonged collar membranelles; PK, posterior kinety; RF, right ciliary field; VK, ventral kineties. Scale bars: 35 μm (**A**), 30 μm (**B**,**C**), 15 μm (**D**,**E**), 20 μm (**F**).

***Tintinnopsis tubuformis* Chiang, 1956** (Figure 4 and Figure 5; Table 1)

**Deposition of voucher material.**

A protargol slide with voucher specimens was deposited in the Laboratory of Protozoology, Institute of Evolution and Marine Biodiversity, Ocean University of China, with the registration number BY202302200201.

**Habitat.** Water Temperature 7 °C.

**Zoobank registration**: LSID: 52D8BF64-09C9-48BD-838D-C7789767F618.

**Morphological Description.**

The tubular lorica was 62–111 μm long and possessed a rounded end and a few posterior intumescentiae, with an opening diameter of 20–29 μm (Figure 4A and Figure 5A–E). The ratio between lorica length and opening diameter was about 2.9–3.2:1. The boundary between bowl and collar was indistinct, while the lorica width was slightly wider than the opening diameter (Figure 4A and Figure 5A–E). No annular striae, pores or windows on lorica surface; lorica wall was heavily agglutinated with mineral grains approximately 1–10 μm in diameter (Figure 4A and Figure 5A–E).

Cell body 30–70 × 15–30 μm in vivo when extended and 66–90 μm × 31–46 μm after protargol staining (Figure 4A and Figure 5A–C). The posterior region of the cell tapered gradually into a peduncle approximately 55–70 μm in length, which was anchored to the base of the lorica (Figure 5A). Two ovoid macronuclei, invisible in living cells, 20–36 × 13–22 μm after protargol staining, with 13–19 μm between anterior-most macronucleus and collar membranelles (Figure 5F). Two ovoid micronuclei about 3–5 μm after protargol staining, located at lateral side of macronuclei (Figure 4C and Figure 5F,G). No tentaculoids, contractile vacuoles, cytopyge, or capsules observed. Colorless and transparent cytoplasm, rotational forward movement around central axis in water (Figure 5A).

Somatic ciliary pattern consisted of ventral, dorsal, and posterior kineties and right, left, and lateral ciliary fields (Figure 4B–D and Figure 5F–L). Ventral kinety 24–37 μm in length, contained 26–38 monokinetids, and exhibited variable spacing between the monokinetids; commencing above the second or third kinety of the right ciliary field, about 2–3 μm posterior to the collar membranelles, curved around the left side of the right ciliary field and then followed a course parallel to the kineties of the lateral ciliary field (Figure 4B,D and Figure 5F,J). Right ciliary field consisted of 7–10 kineties, about 5 μm between adjacent kineties; each kinety with one anterior dikinetid and 3–14 posterior monokinetids; while the first kinety commenced about 2 µm posterior to the others, all remaining kineties began approximately 5–10 µm posterior to the collar membranelles (Figure 4B–D and Figure 5F,J,L). Left ciliary field comprising 7–10 kineties, about 5 μm apart and 8 μm posterior to collar membranelles; each kinety with one anterior dikinetid and 1–8 subsequent monokinetids (Figure 4B–D and Figure 5G,H,J,L). In protargol-stained specimens, all basal bodies in the ciliary fields possessed cilia, with the anterior cilium of each dikinetid measuring about 10 μm and all other cilia measuring approximately 4 μm in length (Figure 5F,H,J). Lateral ciliary field consisted of 17–24 monokineties with kinetid densely arranged; protargol staining revealed cilia approximately 3 μm in length (Figure 4B–D and Figure 5F,J). Dorsal kinety was 62–77 μm long and consisted of 33–47 dikinetids, originating about 3 μm posterior to the collar membranelles and about 8 μm and 9 μm from the right and left ciliary fields, respectively; in protargol-stained specimens, a cilium approximately 10 μm long associated solely with the posterior basal body of each dikinetid (Figure 4C,D and Figure 5G,I). Posterior kinety 24–35 μm long, consisted of 7–12 sparsely arranged monokinetids with a long cilium on each monokinetid about 8–10 μm after protargol staining (Figure 4C,D and Figure 5G,I).

Oral apparatus occupying anterior portion of cell (Figure 4A and Figure 5A). Adoral zone of membranelles consisted of 13–15 collar membranelles, three of which extended into the buccal cavity; polykinetids of membranelles about 15 μm long (Figure 4B–D and Figure 5K). Cilia of membranelles about 10–15 μm long (Figure 4A and Figure 5A). One buccal membranelle inside the buccal cavity, with about 10 μm of base. The endoral membrane comprised a single basal body row, encircling the dorsal side of the buccal cavity and extending to the right side (Figure 4B).

## 4. Discussion

***Tintinnopsis wangi* Nie, 1933**

Tintinnopsis wangi was first discovered in a lake in Nanjing, China, by Nie [37]. The lorica length of the original population ranges from 35 to 61.1 μm. Although the opening diameter was not provided, the illustration suggests a length-to-diameter ratio of approximately 2.2:1, as seen in a typical individual measuring about 30 μm. The lorica comprises two parts: a cylindrical collar bearing 3–6 annular constrictions and an ovate bowl with a rounded base. The morphology of the Weishan Lake population reported in the present study resembles that of the original population, although the lorica length is slightly greater (42–71 μm vs. 35–61.1 μm). Since this variation falls within the range of intraspecific differences, the Weishan Lake population is identified as *T. wangi*. The further population was collected in the lakes of Jiangsu and Nanjing, China [39]. The present population also resembles the Jiangsu and Nanjing populations in lorica features (42–71 μm vs. 42–65 μm in length and 20–29 μm vs. 27–35 μm in opening diameter). The live morphology and ciliary pattern of *T, wangi* was first revealed in present study.

Several freshwater *Tintinnopsis* species reported from Chinese lakes share similar lorica features to those of *T. wangi*, e.g., *T. anhuiensis* Nie, 1933, *T. sinensis* Chiang, 1956, *T. leidiyi* Chiang, 1956, and *T. potiformis* Chiang, 1956. *T. anhuiensis* and *T. sinensis* closely resemble *T. wangi* in lorica morphology, i.e., all possessing a cylindrical collar with annular constrictions and an ovate bowl with a rounded end. Differences are limited to lorica length (25–71 μm in *T. wangi*, 20.2–86 μm in *T. sinensis*, 70–84 μm in *T. anhuiensis*) and the distribution of attached particles, which vary depending on particle adhesion and environmental conditions and thus cannot serve as reliable interspecific diagnostic features [37,39]. Therefore, both *T. anhuiensis* and *T. sinensis* should be considered synonyms of *T. wangi*. In contrast, *T. leidiyi* and *T. potiformis* can be distinguished from *T. wangi* by their flared collars (the collar of *T. wangi* is not flared), and, additionally, *T. leidiyi* exhibits a tapered, inverted conical bowl (whereas that of *T. wangi* is ovate with a rounded end) [39].

It is noteworthy that the posterior kinety of *T. wangi* and *T. tubuformis* are both monokinetids, while all posterior kineties of marine *Tintinnopsis* species with known ciliary patterns are dikineties [28,29,30,31]. This difference may indicate a synapomorphy for freshwater *Tintinnopsis* species, which may be related to the differences in lorica construction between freshwater and marine environments and requires support from molecular data and more ciliature pattern information from freshwater species.

***Tintinnopsis tubuformis* Chiang, 1956**

*Tintinnopsis tubuformis* Chiang, 1956, was first discovered in Wuli Lake, China, by Chiang [39]. The original population closely resembles the Weishan Lake population in its test-tube-shaped lorica, length (50–92 μm vs. 62–111 μm), and absence of annular striae. Although the original population has a slightly larger aperture diameter (29–35 μm vs. 20–29 μm), this difference is also considered intraspecific.

He et al. [30] provided a redescription of a Shanghai population of *Tintinnopsis tubuformis*, including the first report of its ciliary pattern. The ciliary pattern observed in the Weishan Lake population is generally similar to that of the Shanghai population, though the number of somatic kineties is significantly higher (17–24 vs. 11–13). In addition, He et al. [30] also noted considerable variation in lorica length and regarded *Tintinnopsis longa* as a synonym of *T. tubuformis*, which was also obtained by the lorica data in the present study.

## 5. Conclusions

In this study, two freshwater tintinnid ciliates, *Tintinnopsis wangi* and *T. tubuformis*, collected from the Weishan Lake Basin in China, were redescribed utilizing living observation and protargol staining. By integrating lorica features, living morphology, and infraciliature details revealed via protargol staining, the taxonomic diagnosis of *T. wangi* was updated. Notably, the findings also emphasize the intraspecific stability of ciliary patterns within Tintinnida, as the infraciliature of the *T. tubuformis* Weishan Lake population corresponded well with previously reported populations. Interestingly, the discovery that both investigated freshwater species possess monokinetid posterior kineties (in contrast to the dikinetids found in known marine *Tintinnopsis* species) suggests a distinct evolutionary pathway for freshwater tintinnids. Ultimately, this research effectively bridges critical gaps in the morphological database of freshwater ciliates, providing a reliable and essential reference for future phylogenetic analyses, water quality assessment, and lake biodiversity conservation.

## Figures and Tables

**Table 1:** Morphometric data of *Tintinnopsis wangi* and *T. tubuformis* (measurements in μm). Lorica data are based on live specimens, and other data are based on protargol-stained specimens. Abbreviations: CV, coefficient of variation in %; M, median; Max, maximum; Mean, arithmetic mean; Min, minimum; N, number of specimens examined; SD, standard deviation.

Characters	Species	Min	Max	Mean	M	SD	CV	N
Lorica, length	*T. wangi*	42	71	55.6	56	9.5	17.1	9
	*T. tubuformis*	62	111	91.8	96	14.0	15.3	12
Lorica, opening diameter	*T. wangi*	27	34	30.3	31	2.69	8.9	9
	*T. tubuformis*	20	29	23.3	22	2.7	11.6	12
Cell, length	*T. wangi*	47	72	64.1	67	7.62	11.9	15
	*T. tubuformis*	66	90	78.3	79	7.15	9.1	15
Cell, width	*T. wangi*	27	37	32.3	33	3.18	9.8	15
	*T. tubuformis*	31	46	36.5	36	4.36	11.9	15
Macronuclear nodules, length	*T. wangi*	15	19	16.9	17	1.03	6.1	15
	*T. tubuformis*	20	36	26.8	26	4.57	17.1	15
Macronuclear nodules, width	*T. wangi*	10	14	11.9	12	1.22	10.3	15
	*T. tubuformis*	13	22	16.9	17	2.6	15.4	15
Ventral kinety, length	*T. wangi*	17	26	20.1	20	2.66	13.2	15
	*T. tubuformis*	24	37	30.3	30	3.35	11.1	15
Dorsal kinety, length	*T. wangi*	37	52	41.6	41	4.12	9.9	15
	*T. tubuformis*	62	77	69.3	70	5.11	7.4	15
Posterior kinety, length	*T. wangi*	10	15	12.3	12	1.39	11.3	15
	*T. tubuformis*	24	35	31.1	31	2.7	8.7	15
Longest kinety in left field, length	*T. wangi*	9	13	11.6	12	1.06	9.1	15
	*T. tubuformis*	21	27	23.8	24	1.97	8.3	15
Shortest kinety in left field, length	*T. wangi*	2	5	3.5	12	1.05	29.7	15
	*T. tubuformis*	10	14	12.3	12	1.05	8.5	15
Longest kinety in right field, length	*T. wangi*	8	12	9.8	10	1.15	11.7	15
	*T. tubuformis*	26	37	32.1	32	3.2	10.0	15
Shortest kinety in right field, length	*T. wangi*	3	5	4.0	4	0.76	18.9	15
	*T. tubuformis*	10	17	14.3	14	1.91	13.4	15
Lateral ciliary field, number of kineties	*T. wangi*	12	17	15.0	15	1.46	9.7	15
	*T. tubuformis*	17	24	18.7	18	2.23	11.9	15
Collar membranelles, number	*T. wangi*	16	18	17.10	17	0.7	4.1	15
	*T. tubuformis*	13	15	14.10	14	0.52	3.7	15
Prolonged membranelles, number	*T. wangi*	3	3	3.00	3	0	0.0	15
	*T. tubuformis*	3	3	3.00	3	0	0.0	15
Buccal membranelles, number	*T. wangi*	1	1	1.00	1	0	0.0	15
	*T. tubuformis*	1	1	1.00	1	0	0.0	15
Adoral zone of membranelles, diameter	*T. wangi*	24	35	29.80	30	2.81	9.4	15
	*T. tubuformis*	27	37	32.30	32	2.6	8.1	15
Macronuclei, number	*T. wangi*	2	2	2.0	2	0.00	0.0	15
	*T. tubuformis*	2	2	2.0	2	0.00	0.0	15

## Data Availability

The original contributions presented in this study are included in the article. Further inquiries can be directed to the corresponding authors.

## References

[1] Gao F., Bai Y., Chi Y., Feng X.C., Lian C.Y., Lu B.R., Luo X.T., Ma M.Z., Wang C.C., Wang Y.R. (2025). Current status of phylogenetic studies on ciliated protists (Alveolata, Protozoa, Ciliophora) by the OUC group: Advances, challenges and future perspectives. Mar. Life Sci. Technol..

[2] Hu X., Lin X., Song W. (2019). Ciliate Atlas: Species Found in the South China Sea.

[3] Song W.B., Warren A., Hu X.Z. (2009). Free-Living Ciliates in the Bohai and Yellow Seas, China.

[4] Wang R., Bai Y., Hu T., Xu D., Suzuki T., Hu X. (2021). Integrative taxonomy and molecular phylogeny of three poorly known tintinnine ciliates, with the establishment of a new genus (Protista; Ciliophora; Oligotrichea). BMC Ecol. Evol..

[5] Zheng W.B., Li C., Zhou Z.R., Chen X., Lynch M., Yan Y. (2025). Unveiling an ancient whole-genome duplication event in *Stentor*, the model unicellular eukaryotes. Sci. China Life Sci..

[6] Dolan J.R., Pierce R.W., Dolan J.R., Montagnes D.J.S., Agatha S., Coats W.D., Stoecker D.K. (2012). Diversity and distributions of tintinnids. The Biology and Ecology of Tintinnid Ciliates: Models for Marine Plankton.

[7] Santoferrara L.F., Rubin E., Mcmanus G.B. (2018). Global and local DNA (meta) barcoding reveal new biogeography patterns in tintinnid ciliates. J. Plankton Res..

[8] Zhu C., Liu W., Li X., Xu Y., El-Serehy H.A., Al-Farraj S.A., Ma H., Stoeck T., Yi Z. (2021). High salinity gradients and intermediate spatial scales shaped similar biogeographical and co-occurrence patterns of microeukaryotes in a tropical freshwater-saltwater ecosystem. Environ. Microbiol..

[9] Garcia M.D., Barría de Cao M.S. (2018). Anthropogenic pollution along the coast of a temperate estuary: Effects on tintinnid assemblages. Hydrobiologia.

[10] Rakshit D., Sahu G., Mohanty A.K., Satpathy K.K., Jonathan M.P., Murugan K., Sarkar S.K. (2017). Bioindicator role of tintinnid (Protozoa: Ciliophora) for water quality monitoring in Kalpakkam, Tamil Nadu, south east coast of India. Mar. Pollut. Bull..

[11] Jönsson P.R., Johansson M., Pierce R.W. (2004). Attachment to suspended particles may improve foraging and reduce predation risk for tintinnid ciliates. Limnol. Oceanogr..

[12] Zingel P., Agasild H., Karus K., Buholce L., Nõges T. (2019). Importance of ciliates as food for fish larvae in a shallow sea bay and a large shallow lake. Eur. J. Protistol..

[13] Dunthorn M., Lipps J.H., Dolan J.R., Abboud-Abi Saab M., Aescht E., Bachy C., Barría de Cao M.S., Berger H., Bourland W.A., Choi J.K. (2015). Ciliates-Protists with complex morphologies and ambiguous early fossil record. Mar. Micropaleontol..

[14] Lipps J.H., Stoeck T., Dunthorn M., Dolan J.R., Montagnes D.J.S., Agatha S., Coats W.D., Stoecker D.K. (2013). Fossil tintinnids. The Biology and Ecology of Tintinnid Ciliates: Models for Marine Plankton.

[15] Brandt K. (1906). Die Tintinnodeen der Plankton Expedition. Tafelerklärungen nebst kurzer Diagnose der neuen Arten. Ergebnisse der Plankton-Expedition der Humboldt-Stiftung.

[16] Kofoid C.A., Campbell A.S. (1929). A conspectus of the marine and fresh-water Ciliata belonging to the suborder Tintinnoinea, with descriptions of new species principally from the Agassiz Expedition to the Eastern Tropical Pacific 1904–1905. Univ. Calif. Publ. Zool..

[17] Kofoid C.A., Campbell A.S. (1939). Reports on the scientific results of the expedition to the Eastern Tropical Pacific, in charge of Alexander Agassiz, by the U. S. Fish Commission Steamer “Albatross,” from October, 1904, to March, 1905, Lieut. Commander L M Garrett, U S N Commanding XXXVII. The Ciliata: The Tintinnoinea. Bull. Mus. Comp. Zool..

[18] Zhang W.C., Feng M.P., Yu Y., Zhang C.X., Xiao T. (2012). An Illustrated Guide to Contemporary Tintinnids in the World.

[19] Laval-Peuto M. (1981). Construction of the lorica in Ciliata Tintinnina. In vivo study of *Favella ehrenbergii*: Variability of the phenotypes during the cycle, biology, statistics, biometry. Protistologica.

[20] Agatha S., Strüder-Kypke M.C., Dolan J.R., Montagnes D.J.S., Agatha S., Coats W.D., Stoecker D.K. (2013). Systematics and evolution of tintinnid ciliates. The Biology and Ecology of Tintinnid Ciliates: Models for Marine Plankton.

[21] Santoferrara L.F., Bachy C., Alder V.A., Gong J., Kim Y.O., Saccà A., Silva Neto I.D.D., Strüder-Kypke M.C., Warren A., Xu D.P. (2016). Updating biodiversity studies in loricate protists: The case of the tintinnids (Alveolata, Ciliophora, Spirotrichea). J. Eukaryot. Microbiol..

[22] Agatha S. (2010). Redescription of *Tintinnopsis parvula* Jörgensen, 1912 (Ciliophora: Spirotrichea: Tintinnina), including a novel lorica matrix. Acta Protozool..

[23] Bai Y., Wang R., Al-Rasheid K., Miao M., Hu X.Z. (2020). The type species of *Amphorellopsis* and *Tintinnopsis* (Protozoa: Ciliophora): A new ciliary pattern and some comments in Tintinnina. J. King Saud Univ. Sci..

[24] Bai Y., Wang R., Liu W., Warren A., Zhao Y., Hu X.Z. (2020). Redescriptions of three tintinnine ciliates (Ciliophora: Tintinnina) from coastal waters in China based on lorica features, cell morphology, and rDNA sequence data. Eur. J. Protistol..

[25] Bai Y., Wang R., Song W., Li L., Hu X.Z. (2020). Three redescriptions in *Tintinnopsis* (Protista: Ciliophora: Tintinnina) from coastal waters of China, with cytology and phylogenetic analyses based on ribosomal RNA genes. BMC Microbiol..

[26] Gruber M.S., Strüder-Kypke M., Agatha S. (2018). Redescription of *Tintinnopsis everta* Kofoid and Campbell, 1929 (Alveolata, Ciliophora, Tintinnina) Based on Taxonomic and Genetic Analyses–Discovery of a New Complex Ciliary Pattern. J. Eukaryot. Microbiol..

[27] Jiang Y., Yang J.P., Al-Farraj S., Warren A., Lin X.F. (2012). Redescriptions of three tintinnine ciliates, *Tintinnopsis tocantinensis*, *T. radix*, and *T. cylindrica* (Ciliophora, Spirotrichea), from coastal waters off China. Eur. J. Protistol..

[28] Foissner W., Berger H., Schaumburg J. (1999). Identification and Ecology of Limnetic Plankton Ciliates.

[29] Foissner W., O’Donoghue P.J. (1989). Morphology and infraciliature of some freshwater ciliates (Protozoa: Ciliophora) from Western and South Australia. Invert. Syst..

[30] He J., Jiang J., Agatha S., Pan H. (2022). Taxonomy and phylogeny of the freshwater tintinnid *Tintinnopsis tubuformis* Chiang, 1956 (Ciliophora, Oligotrichea) and a proposed synonymization of *T. longa* nom. corr. Chiang, 1956. J. Eukaryot. Microbiol..

[31] Song W.B., Wilbert N. (1989). Taxonomische Untersuchungen an Aufwuchsciliaten (Protozoa, Ciliophora) im Poppelsdorfer Weiher, Bonn. Lauterbornia.

[32] Stein F. (1867). Der Organismus der Infusionsthiere Nach Eigenen Forschungen in Systematischer Reihenfolge Bearbeitet.

[33] Pan X.M., Bourland W., Song W.B. (2013). Protargol Synthesis: An In-house Protocol. J. Eukaryot. Microbiol..

[34] Wilbert N. (1975). Eine Verbesserte Technik der Protargolimprägnation für Ciliaten. Mikrokosmos.

[35] Agatha S., Riedel-Lorjé J.C. (2006). Redescription of *Tintinnopsis cylindrica* Daday, 1887 (Ciliophora: Spirotricha) and unification of tintinnid terminology. Acta Protozool..

[36] Santoferrara L.F., Alder V.V., McManus G.B. (2017). Phylogeny, classification and diversity of Choreotrichia and Oligotrichia (Ciliophora, Spirotrichea). Mol. Phylogenet. Evol..

[37] Nie D.S. (1933). Notes on three new species of fresh-water tintinnoinea. Sinensia.

[38] ICZN (International Commission on Zoological Nomenclature) (1999). International Code of Zoological Nomenclature.

[39] Ciang S.C. (1956). Notes on the freshwater tintinnoinea from Kingsu and Anhui provinces. Acta Hydrobiol. Sin..

